# Effect of Yeast Polysaccharides Replacing Sulfur Dioxide on Antioxidant Property and Quality Characteristics of *Cabernet Sauvignon* Wines

**DOI:** 10.3390/foods14183188

**Published:** 2025-09-12

**Authors:** Rui Liao, Xiongjun Xiao, Huiling Huang, Kangjie Yu, Jianxia Tan, Yue Wang, Cong Li, Siyu Li, Yi Ma

**Affiliations:** 1 College of Bioengineering, Sichuan University of Science and Engineering, Yibin 644000, China; 323095102306@stu.suse.edu.cn (R.L.); 18828929169@163.com (X.X.); huiling20000818@163.com (H.H.); 323095102321@stu.suse.edu.cn (J.T.); 323086002220@stu.suse.cn (Y.W.); 323086002212@stu.suse.edu.cn (C.L.); 324095102420@stu.suse.edu.cn (S.L.); 2 Engineering Technology Research Center of Special Grain for Wine Making, Yibin 644000, China; yukj@nwafu.edu.cn; 3 State Key Laboratory for Crop Stress Resistance and High-Efficiency Production, College of Agronomy, Northwest A&F University, Xianyang 712100, China

**Keywords:** *Cabernet Sauvignon* wines, SO_2_ substitution, yeast polysaccharides, volatiles

## Abstract

This study investigated the potential of yeast polysaccharides (YPs) as an SO_2_ substitute by adding varying concentrations (0–250 mg/L) prior to wine fermentation. The antioxidant activity and color stability were evaluated using UV–visible spectrophotometry, high-performance liquid chromatography, and colorimetric methods. Furthermore, HS-SPME-GC-MS and quantitative descriptive analysis were employed to compare the volatile compound profiles and sensory attributes between the optimal yeast polysaccharide treatment group (TS100, 100 mg/L) and the sulfur dioxide treatment group (S). Results showed that YPs addition effectively preserved phenolic compounds, with TS100 exhibiting the highest total phenols, flavonoids, and anthocyanins, where total anthocyanins reached 340.79 mg/L, a 2.52% increase over the S group. ABTS^+^ scavenging activity showed no significant difference from the S group, confirming strong antioxidant capacity. Flavor compound content was higher in TS100 (10,458.99 μg/L) than in the S group (10,156.07 μg/L), with ethyl caprylate and ethyl decanoate being particularly prominent. These esters contributed intense fatty and fruity aromas, enhancing the overall sensory quality of the wine. In summary, 100 mg/L YPs addition yielded the best wine quality, demonstrating promising potential as an SO_2_ alternative in winemaking.

## 1. Introduction

Sulfur dioxide (SO_2_) is widely applied in the wine industry due to its outstanding antimicrobial and antioxidant properties, effectively preventing oxidative browning and microbial spoilage [1]. However, increasing evidence has raised concerns regarding its potential health risks. SO_2_-containing wines have been reported to cause allergic reactions in sensitive individuals [2,3]. Moreover, SO_2_-bioaccumulation has been linked to an elevated risk of lung cancer [4,5]. Considering growing global attention to food additive safety, identifying effective alternatives to SO_2_ or strategies to reduce its usage has become a pressing issue.

Previously, we have used polyphenols to replace SO_2_ in wine making, and the results showed that dihydromyricetin has a good antioxidant capacity, resveratrol contributes the most to the wine aroma, and catechins are optimal for the sensory characteristics of the wine [6]. However, they cannot completely replace the role of SO_2_, and suitable phenolic antioxidants need to be selected according to specific production requirements. Current strategies for reducing SO_2_ in wine include the use of lysozyme, glutathione, and non-thermal sterilization techniques (including ultrasonic, ultrahigh pressure, and pulsed electric fields), among others [7,8,9,10,11,12]. Although these methods can partially replace the antioxidant and antimicrobial functions of SO_2_, they still suffer from negative problems such as lack of stability and high cost [13]. A method that can completely replace SO_2_ addition in industrial production has yet to be found.

Yeast polysaccharides are macromolecular polysaccharide complexes derived from yeast cells, which are mainly composed of β-glucan, mannan, and a small amount of chitin [14]. These components possess antioxidant properties, enabling them to scavenge free radicals and suppress lipid peroxidation [15,16,17]. During the beer production process, the rapid growth of yeast can increase its biomass by approximately 3 to 5 times [18]. It contains a large amount of untapped yeast cell wall polysaccharides, most of which are discharged as waste [19]. The utilization of yeast polysaccharides is of great importance for improving resource utilization and environmental protection. Studies have shown that yeast polysaccharides can improve the antioxidant capacity of food, the immunity of livestock, and have a certain inhibitory adsorption effect on pathogenic bacteria [20,21]. Moreover, they are derived from abundant sources, exhibit low residue levels, and are considered non-toxic with no known side effects [14]. Rinaldi et al. [22] carried out a study on the effect of polysaccharides on the stability of wine. Yeast polysaccharides can enhance the body of the wine flowers and fruits, and improve the aroma and taste, significantly enhancing the quality of the wine [23]. Therefore, we speculate that yeast polysaccharides may serve as a substitute for SO_2_ by virtue of their antioxidant and antimicrobial properties, helping to maintain the stability and sensory quality of wine. However, the antioxidant properties of yeast polysaccharides in wines and their effects on the organoleptic properties of wines have not been sufficiently reported yet.

In this study, yeast polysaccharides were used in *Cabernet Sauvignon* wines at different concentrations to completely replace SO_2_. And their effects on the physicochemical indexes, antioxidant activity, color stability, and organoleptic qualities of the wines were evaluated and compared with those of the wines with traditional addition of SO_2_. The aim was to explore the potential application of yeast polysaccharides in sulfur-free winemaking and to provide basis and technical support for the development of healthy and safe wine products.

## 2. Materials and Methods

### 2.1. Materials and Reagents

*Cabernet Sauvignon* grapes were purchased from Zhangjiakou Dahaoheshan Brewing Co., Ltd. (Zhangjiakou, China), and the grapes were harvested in 2024. Yeast polysaccharides were purchased from Shanghai McLean Biochemical Science and Technology Co., Ltd. (Shanghai, China). Fruit wine-making yeast was purchased from Angel Yeast Co., Ltd. (Yichang, China). Potassium metabisulphite, sucrose, phenolphthalein, and tartaric acid were analytically pure from Shanghai Aladdin Biochemical Science and Technology Co., Ltd. (Shanghai, China). Hydrochloric acid, anhydrous ethanol, methanol, aluminum nitrate, Folarin-Danis test solution, rutin, gallic acid, and Copper sulfate were analytically pure from Chengdu Cologne Chemical Co., Ltd. (Chengdu, China). The aroma compound standard, C7–C24 normal paraffin standards, and phenolic standards used in this study were all purchased from Jinshudu Laboratory Equipment Co., Ltd. (Chengdu, China).

### 2.2. Wine-Making Operations

The vineyard is located at 40.3° N latitude, which is widely recognized as a prime region for grape cultivation. The grapes were harvested in 2024, a year with relatively low rainfall, resulting in good grape health and ripeness. Grapes were selected with a sugar content of approximately 22 °Brix, a diameter of 12–14 mm, and a deep purple color, while any damaged or diseased berries were removed. The grapes were then destemmed, washed, and crushed. 10 L of grape juice were poured into a 15-L stainless steel fermentation tank, and 60 mg/L of pectinase was added. Different antioxidants were then added to the fermentation tanks (As specified in Section 2.3). The mixture was cold-soaked at 4 °C for 24 h. Afterward, a specific amount of white granulated sugar and citric acid was added to achieve a sugar content of 20 °Brix and a pH of 4.0, respectively. Then, 2.0 g of wine yeast was added to 30 mL of sucrose solution with a sugar content of 5%. This yeast activation solution was placed in a 30 °C water bath for 30 min for activation. After activation, 8% of the yeast suspension was added to the grape juice and stirred gently to increase the dissolved oxygen content. The stainless steel fermentation tank was then maintained at 26 °C for 7 days. After fermentation, the wine was filtered, bottled, and stored at a controlled temperature of 14 °C for two months before analysis.

### 2.3. Test Setup

The fermentation parameters of the experimental groups were set as follows: starting sugar level of 20 °Brix, fermentation temperature of 26 °C, fermentation pH of 4.0, fermentation cycle of 7 days, inoculated yeast amount of 8%, and added 60 mg/L pectinase, according to the amount of added yeast polysaccharides of 0, 50, 100, 150, 200, 250 mg/L, and the set of groups were TS0, TS50, TS100, TS150, TS200, and TS250. Three parallel groups were made for each group. Traditional process group: initial sugar level of 20 °Brix, fermentation temperature of 26 °C, fermentation pH of 4.0, fermentation cycle of 7 days, inoculated yeast amount of 8%, added 60 mg/L pectinase, added potassium metabisulfite 60 mg/L, set up as S group, and do three parallel groups.

### 2.4. Physicochemical Analysis

Soluble solids, reducing sugars, pH, alcohol content, and total acidity were determined according to the experimental methods in GB/T15038-2006 (General Methods of Analysis for Wine and Fruit Wine) [24].

### 2.5. Antioxidant Capacity

The DPPH scavenging rate was referred to the method of Diao et al. [25]. Distilled water was used for zeroing, test sample: 0.1 mL of sample + 3.9 mL of DPPH solution; blank sample: 0.1 mL of distilled water + 3.9 mL of DPPH solution, and it was left to stand for 30 min away from light, and the A_517_ was measured.

(1) $$DPPH\;radical\;scavenging\;activity\left(\%\right)=\frac{A\;control-A\;sample}{A\;control}\times 100\%$$

The ABTS cation radical scavenging rate was determined by referring to the method of Liang et al. [26]. Pipette 1 mL of the sample to be tested with 3 mL of ABTS cation radical working solution, mixed for 10 s, and immediately placed in the dark to react for 6 min, the absorbance was measured at 734 nm (A sample). At the same time, an equal amount of distilled water was used to replace the sample as a blank control test (A control). The ABTS cation radical scavenging rate was calculated according to Formula (2).

(2) $$ABTS\;radical\;scavenging\;activity\left(\%\right)=\frac{A\;control-A\;sample}{A\;control}\times 100\%$$

### 2.6. Determination of Phenolic Substances

The total phenol content was determined by the method of Ma et al. [6] using the Folin-Ciocalteu method, calculated as the mass concentration of gallic acid. 1 mL of sample was mixed and reacted with an equal volume of Folin-Ciocalteu reagent for 3 min, and 1.5 mL of 7.5% mass fraction sodium carbonate solution was added. The A_760_ was determined by protecting the sample from light for 2 h. The standard curve was established as y = 0.1009x + 0.0063, where y is the absorbance, x is the content of gallic acid, and R^2^ = 0.9995.

The total flavonoid content was determined using the rutin-methanol colorimetric method, and the results were expressed as rutin equivalents [27]. The standard curve was plotted: y = 0.0057x + 0.0009, where y is the absorbance, x is the flavonoid content, and R^2^ = 0.9991.

Determination of total anthocyanins content The method of Ma et al. [28] was used. Determination of tannin content Determination of tannin content was carried out in accordance with the national standard GB/T15038-2006 (General Methods of Analysis for Wine and Fruit Wine) [24].

### 2.7. Quantitative Analysis of Phenolic Compounds

Following the method of Wei et al. [29], phenolic compounds in wine were quantitatively analyzed using a Shimadzu LC-2030 HPLC system (Shimadzu, Japan). The chromatographic column was an Agilent ZORBAX SB-Aq (250 mm × 4.6 mm, 5 μm) (Agilent Technologies, Palo Alto, CA, USA). Column temperature: 30 °C. Flow rate: 1.0 mL/min. Injection volume: 20 μL. Detection wavelengths: 280 nm, 320 nm, or 360 nm. The mobile phase consisted of 2% acetic acid (A) and 100% methanol (B). The elution gradient was as follows: 0–10 min, 5–30% B. 10–35 min, 30–50% B. 35–40 min, 50–60% B. 40–45 min, 60–70% B. 45–50 min, 70–5% B. 50–55 min, 5% B.

### 2.8. Color Measurement

Colorimetry was determined with reference to the method of Strati et al. [30] using a CR-400 colorimeter (Konica Minolta Co., Ltd., Tokyo, Japan), calibrated using distilled water.

### 2.9. HS-SPME-GC-MS

Volatile compounds were determined for S and TS100. The analysis was carried out using a 7890A gas chromatograph from Agilent Technologies Co., Ltd. (Beijing, China). The method of Xie et al. [31] was referred to with slight modifications. 8.0 mL of wine and 1 g of NaCl were accurately added into a 15 mL headspace vial, followed by 20 µL of 2-octanol internal standard (0.458 mg/mL). The wine sample was preheated at 45 °C for 10 min. The microextraction head was inserted into the headspace vial. The fiber head (50/30 μm DVB/CAR/PDMS fiber, Supelco Co., Ltd., Bellefonte, PA, USA) was pushed out and adsorbed in the headspace position for 35 min, then the fiber head was withdrawn and sent to the gas chromatography delivery port, and thermally desorbed at 250 °C for 3 min. The chromatographic conditions were as follows: DB-WAX column (60 m × 0.25 mm × 0.25 µm), non-split flow injection method was adopted, and the initial temperature was controlled at 40 °C. The initial temperature was controlled at 40 °C for 5 min, then the temperature was increased to 60 °C at 2 °C/min, then to 180 °C at 5 °C/min, and maintained for 5 min, and then to 230 °C at 10 °C/min with 10 min retention; the flow rate of the carrier gas, high-purity helium, was controlled to be 1.2 mL/min. The mass spectrometry was performed under the conditions of electron impact ionization (EI) with an electron energy of 70 eV, full scanning mode, scanning range of 35–500 amu, and an ion source temperature of 230 °C. The mass spectra of aroma compounds were compared with those in the NIST14 mass spectral library, and compounds with a positive match score greater than 85% were selected. Retention indices (RIs) were calculated using C7–C24 saturated n-alkanes as reference standards, and the obtained RI values were compared with those listed on the retention index database (https://webbook.nist.gov/chemistry (accessed on 10 March 2025)) for qualitative confirmation. Quantification of volatile compounds was carried out by calculating the mass concentration of each component based on the ratio of the peak area of each compound to that of the internal standard.

### 2.10. Wine Sensory Tasting

Sensory evaluation was conducted for S and TS100. Prior to the experiment, 20 volunteers (10 males and 10 females, including 4 national-level fruit wine judges) with experience in sensory evaluation were recruited. According to the method described in GB/T 16291.1-2012 [32], these volunteers underwent basic olfactory tests, aroma matching tests, and descriptive ability assessments in a sensory analysis laboratory. Based on the results, six panelists were selected to form the sensory evaluation panel. After training, the panelists performed olfactory evaluations on the wine samples, which were randomly coded and presented in a randomized order. Each panelist evaluated each sample three times. Subsequently, the sensory panel discussed, developed, eliminated, and integrated aroma descriptors, ultimately forming a consensus vocabulary of aroma attributes (as shown in Table 1). Based on this vocabulary, panelists rated the intensity of each descriptor for each sample on a 9-point scale (1–3 for weak, 4–6 for moderate, 7–9 for strong).

### 2.11. Statistical Analysis

Analyses were performed using SPSS 17.0 software, significance analyses were performed using Duncan’s multiple comparisons method, and orthogonal partial least squares discriminant analysis (OPLS-DA) was analyzed by MetaboAnalyst (https://www.metaboanalyst.ca/ (accessed on 14 April 2025)). Principal component analysis (PCA) was analyzed by Simca 14.1. The images were plotted using Origin 2025, except for the evaluator repeatability and stability tests, which were performed using Panel Check 1.4.2 software.

## 3. Results

### 3.1. Effect of Yeast Polysaccharides on the Physicochemical Indices of Wine

The physicochemical indexes of the wines were determined and the results are shown in Table 2. Relative to the control group (TS0), the treatment group with added yeast polysaccharides showed a reduced level of reducing sugars, indicating that the polysaccharide addition modulated yeast growth, facilitated sugar utilization, and expedited the fermentation process [33]. Among them, the TS100 group with the addition of 100 mg/L YPs had the lowest content of reducing sugar and soluble solids, which was close to that of the S group, indicating that the growth activity of brewing yeast was the most vigorous at this concentration, and thus the alcohol content of wines fermented at this concentration was higher than that of the other groups. Yeast polysaccharides were able to significantly reduce the acidity of the wines [34], and there was no significant difference in pH between the groups (*p* > 0.05).

### 3.2. Antioxidant Activity of Wines

**Figure 1:**
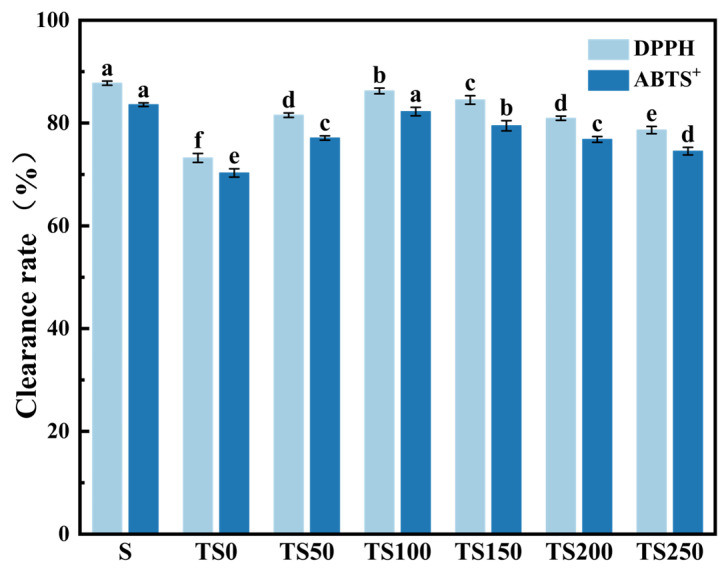
In vitro antioxidant activity of *Cabernet Sauvignon* wines from different treatment groups. Different superscript letters (a–f) for the same parameter denote significant differences (*p* < 0.05).

The in vitro antioxidant activities of *Cabernet Sauvignon* wines fermented under different treatment conditions are shown in Figure 1. Compared with the wines without added yeast polysaccharides, the DPPH and ABTS^+^ radical scavenging rates of the wines treated with added yeast polysaccharides were significantly increased (*p* < 0.05). With the increasing addition of yeast polysaccharides, the antioxidant activity of the wine showed a trend of first increasing and then decreasing. Among them, the TS100 group (100 mg/L YPs) exhibited the most significant improvement in antioxidant capacity, with a DPPH radical scavenging rate reaching 86.25%, an increase of 13.04% compared to the TS0 group. The ABTS^+^ radical scavenging rate was 82.23%, 16.99% higher than that of the TS0 group, and showed no significant difference from the wine fermented with traditional sulfur dioxide addition.

### 3.3. Effect of Yeast Polysaccharides on the Phenolic Content of Cabernet Sauvignon Wines

#### 3.3.1. Polyphenols

**Figure 2:**
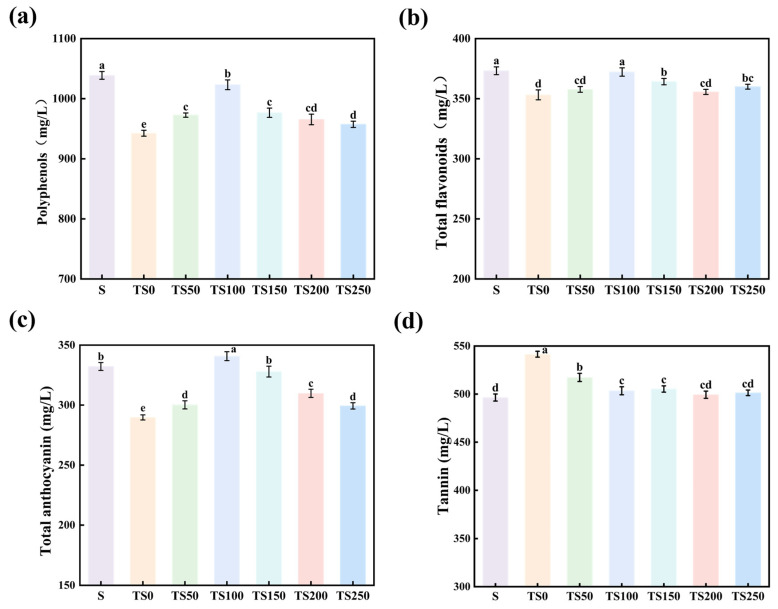
Wine phenolic content. (**a**) Polyphenols; (**b**) Total flavonoids; (**c**) Total anthocyanin; (**d**) Tannin. Data points represent the mean values of three parallel replicates. Different superscript letters (a–e) for the same parameter denote significant differences (*p* < 0.05).

Polyphenols have a great influence on the color, taste, and antioxidant property of wine [35], and the polyphenols content of wines in different treatment groups is shown in Figure 2a. The polyphenols content of the wines in each experimental group was significantly higher than that of the TS0 group (*p* < 0.05), indicating that both yeast polysaccharides and SO_2_ can effectively inhibit the oxidation reaction of wine phenolics and reduce the loss of polyphenols. With the increase of yeast polysaccharide addition, the polyphenols content of wine showed a trend of increasing and then decreasing. The TS100 group had the highest polyphenols content of 1023.16 mg/L, which was 11.64% higher than that of the group without yeast polysaccharide addition, close to that of the SO_2_-treated S group. Subsequently, with the increasing addition of yeast polysaccharides, the polyphenols content decreased. This trend of an initial increase followed by a decline in polyphenols may be attributed to the encapsulating effect of yeast polysaccharides on phenolic compounds in the liquor, which can protect them from oxidation or slow down oxidative browning [36,37,38,39]. However, excessive yeast polysaccharides may form a network structure that traps phenolic compounds, leading to their coprecipitation and thereby resulting in a reduction in the measured polyphenols content.

#### 3.3.2. Total Flavonoids

The wines with SO_2_ and different concentrations of yeast polysaccharides were subjected to the determination of total flavonoid content, and the results are shown in Figure 2b. In the yeast polysaccharide-supplemented groups, the total flavonoid content showed an initial increase followed by a decrease with increasing polysaccharide addition. The TS100 group exhibited the highest total flavonoid content, reaching 372.19 mg/L, which was significantly higher than that of the other yeast polysaccharide-treated groups (*p* < 0.05), and not significantly different from the S group. When the addition exceeded 100 mg/L, the total flavonoid content decreased. These results suggest that an appropriate amount of yeast polysaccharides can enhance the stability or release of flavonoids in the liquor, while higher concentrations exert an inhibitory effect. This may be due to the generation of peroxidative radicals from the oxidation of antioxidant components, which possess oxidative activity and can trigger a chain of adverse side reactions, ultimately leading to a reduction in flavonoid content [25].

#### 3.3.3. Total Anthocyanin

As shown in Figure 2c, the total anthocyanin content in wines with added yeast polysaccharides was significantly higher than that in the TS0 group (*p* < 0.05). This may be attributed to the release of high-molecular-weight mannoproteins by yeast polysaccharides, which aggregate with monomeric anthocyanins in the wine to form stable polymeric complexes, thereby significantly increasing the levels of anthocyanins and phenolic acids. When the YPs concentration was 100 mg/L, the total anthocyanin content in the TS100 group reached its peak at 340.79 mg/L, which was 17.62% higher than that of the TS0 group and 2.52% higher than that of the S group, showing a significant increase compared with the S group (*p* < 0.05). Yeast polysaccharides primarily interact with anthocyanins through non-covalent and hydrogen bonds, forming vertically or horizontally stacked complexes that enhance the stability of anthocyanins in wine [40]. However, high concentrations of yeast polysaccharides may increase the viscosity of the system, hinder the dispersion of anthocyanins, and thus reduce their apparent content.

#### 3.3.4. Tannin

Tannin content has an important effect on the sensory color stability of wine, and Figure 2d demonstrates the effect of different yeast polysaccharide additions on the tannin content of wine. The results showed that the TS0 group without SO_2_ and yeast polysaccharide addition had the highest tannin content, reaching 541.49 mg/L, significantly higher than all other groups (*p* < 0.05). With the increase of yeast polysaccharide addition, the tannin content in wine showed a decreasing trend, which gradually leveled off when the addition amount reached 100 mg/L. This may be due to the fact that yeast polysaccharides bind to tannins through hydrogen bonding, hydrophobic interactions, or electrostatic interactions to form soluble complexes or insoluble precipitates [41], which reduces the detection value of free tannins. In wine, anthocyanins can combine with tannins through covalent and non-covalent interactions to form anthocyanin–tannin polymers. This process may reduce the concentration of free anthocyanins [42]. However, yeast polysaccharides may preferentially bind to tannins via hydrogen bonding and hydrophobic interactions [43]. Tannins bound by YPs are unable to freely participate in condensation reactions, thereby reducing their consumption in forming stable polymers with anthocyanins; consequently, the proportion of free anthocyanins increases [44].

### 3.4. Analysis of Phenolic Compounds

**Figure 3:**
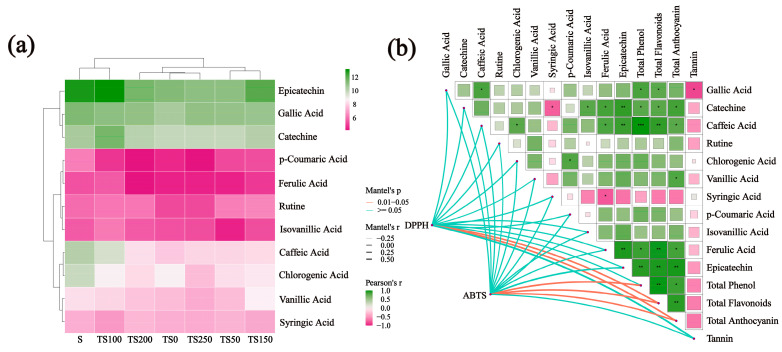
(**a**) Phenolic compounds content in wine. (**b**) Correlation network heatmap of total phenols, total flavonoids, total anthocyanins, tannins, and phenolic compounds. In the heatmap, thicker lines indicate stronger correlations, and colors represent the degree of correlation. Green and purple asterisks denote positive correlation and negative correlation, respectively (paired-sample *t*-test: * *p* < 0.05; ** *p* < 0.01; *** *p* < 0.001).

In this study, HPLC was used to quantitatively analyze 11 common phenolic compounds in different types of wine. A heatmap visually displays the phenolic content under various treatments (Figure 3a). The results showed that the addition of YPs altered the phenolic profile of the wines. Epicatechin and catechin contents were highest in the TS100 group, indicating that an appropriate amount of yeast polysaccharides can effectively preserve the major flavanols. Some researchers have suggested that flavanols in wine are prone to precipitation, while YPs can protect flavanols from precipitation through steric hindrance and stabilize their interactions with anthocyanins [45]. The levels of phenolic acids such as p-coumaric acid and chlorogenic acid decreased with yeast polysaccharide addition. Caffeic acid and p-coumaric acid were significantly lower in all YPs groups than in the S group (*p* < 0.05), suggesting that YPs offer weaker protection than SO_2_. When the YPs dosage was over 200 mg/L, the contents of vanillic acid and ferulic acid decreased, which is consistent with the colloidal flocculation phenomenon caused by excessive polysaccharides [41].

The correlation network heatmap shown in Figure 3b was used to explore the relationship between changes in antioxidant properties and differences in phenolic compound contents. A statistically significant positive correlation (*r* > 0.5) was observed between total flavonoid contents and the DPPH and ABTS radical scavenging capacities (*p* < 0.05), the same as total anthocyanin. In addition, a positive correlation (*r* > 0.5) was found between total phenolic content and ABTS radical scavenging capacity (*p* < 0.05). Although monomeric phenols have a beneficial effect on the antioxidant properties of wine, they were not the main factors contributing to the variation in antioxidant activity observed in this study. The changes in antioxidant capacity were attributed to quantitative variations in phenolic contents.

### 3.5. Effect of Yeast Polysaccharides on the Color Index of Cabernet Sauvignon Wines

**Figure 4:**
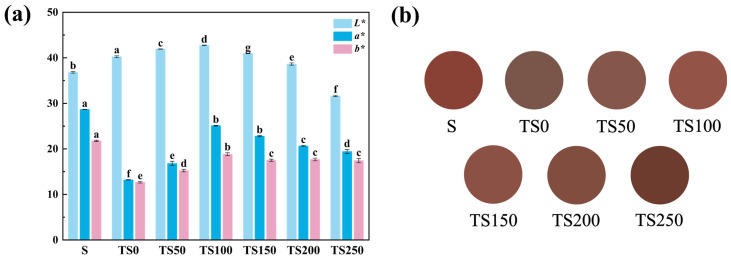
Color difference. (**a**) Color difference between groups of samples at the end of fermentation; (**b**) Characteristic color diagram of wine samples. Different superscript letters (a–g) for the same parameter denote significant differences (*p* < 0.05).

Color is a key parameter in evaluating wine quality. Figure 4a presents the differences in lightness (L*) and chromatic coordinates (a*, b*) among wines from different treatment groups. All samples showed lower L* values than the TS0 group, while both a* and b* values were significantly higher, indicating that the addition of yeast polysaccharides contributed to color stabilization in wine. This effect may be attributed to the negatively charged mannose phosphate groups in YPs can interact electrostatically and ionically with other positively charged substances in the wine [46], thereby hindering pigment precipitation and slowing down pigment oxidation [47], which helps maintain the color stability of the wine.

To visualize the color differences more intuitively, the CIELAB was used to plot the chromatic variation, as shown in Figure 4b. The S group had the highest a* value, displaying the typical purplish-red hue of conventional red wine. Among the yeast polysaccharide-treated groups, TS50 exhibited the highest lightness (L*) but had relatively lower a* and b* values, resulting in the color slightly deviating from the purplish-red tone of red wine. TS100 and TS150 showed no significant difference in a* values, both of which were higher than those of the other yeast polysaccharide groups, indicating that their color tended more toward red and was visually superior. The TS200 and TS250 groups had the darkest wine color, with hues leaning toward grayish brown. This may be due to the excessive addition of yeast polysaccharides indirectly increasing the concentration of free phenolics, which are oxidized into quinones and further polymerized into brown or yellowish-brown high-molecular-weight pigments [35]. Among all yeast polysaccharide-treated groups, TS100 displayed the most favorable color characteristics, most closely resembling that of the SO_2_-treated group.

### 3.6. Flavor and Sensory Evaluation

Based on a comprehensive evaluation of the physicochemical properties, polyphenols, total flavonoids, total anthocyanins, total tannins, and antioxidant activity of wines produced with different levels of yeast polysaccharide addition, the TS100 group was identified as the optimal treatment. It is presumed to have the highest potential to serve as an alternative to traditional SO_2_-treated wine. Therefore, further comparison of volatile compounds and sensory attributes was conducted between the TS100 and S groups.

#### 3.6.1. Analysis of Volatile Substances in Wines

**Figure 5:**
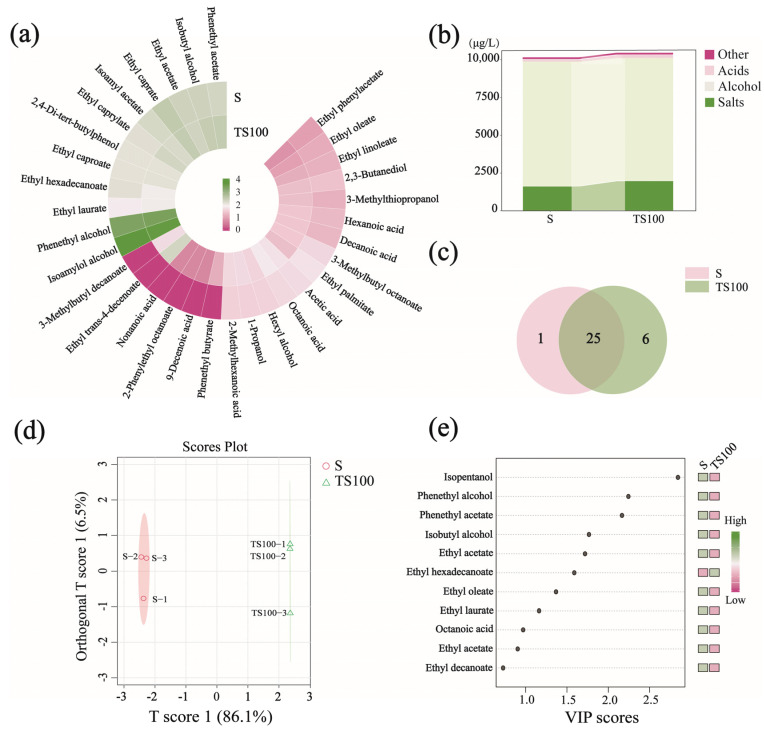
Wine Volatiles. (**a**) Heat map of volatile matter content of two groups of wines; (**b**) Stacked plot of volatile matter categories of wines; (**c**) Venn diagrams of volatile substances in two groups of wine; (**d**) Score of OPLS-DA samples for wine aroma; (**e**) VIP values of key aroma components.

Quantitative analysis of volatile compounds was conducted for the SO_2_-treated (S) and yeast polysaccharide-treated (TS100) wine samples. The data were processed using logarithmic transformation, and the volatile aroma compound contents in both groups are presented in Figure 5a–c. A total of 32 volatile compounds were detected across the two groups: 26 in the S group (total content: 10,156.07 μg/L) and 31 in the TS100 group (total content: 10,458.99 μg/L), with 7 aroma compounds differing between the two groups (Table S1). The wine treated with yeast polysaccharides exhibited a higher content of aroma compounds compared to the SO_2_-treated wine, suggesting that yeast polysaccharides have a positive effect on aroma preservation [48]. This may be attributed to their ability to protect volatile compounds through adsorption, reduce surface tension to promote their release, and ultimately enhance the amount and intensity of perceivable aroma components.

The total alcohol content was higher in the S group (8263.6 μg/L) than in the TS100 group (8146.73 μg/L). Isoamyl alcohol and phenyl alcohol were the predominant alcohols in both wines. Notably, the TS100 group (2893.31 μg/L) had a higher phenyl alcohol content than the S group (2505.83 μg/L); phenyl alcohol imparts distinctive rose and honey-like aromas to wine [49]. Conversely, isoamyl alcohol was more abundant in the S group, contributing medicinal and resin-like notes. The concentration of isobutyl alcohol in the TS100 group was 321.59 μg/L, significantly higher than that in the S group (252.43 μg/L). isobutyl alcohol is a byproduct of branched-chain amino acid metabolism via the Ehrlich pathway during yeast fermentation [50]. The lower isobutyl alcohol content in the S group may be attributed to the inhibitory effect of SO_2_ on microbial activity, which potentially suppressed yeast metabolism and limited Ehrlich pathway activity.

Esters contribute significantly to the aroma of wine, and their total content was higher in the TS100 group (2097.87 μg/L) than in the S group (1612.9 μg/L), indicating that yeast polysaccharides have a positive effect on the production of esters in wine [51]. In the TS100 group, the concentrations of ethyl caprylate (297.63 μg/L), ethyl caprate (392.96 μg/L), and ethyl trans-4-decenoate (222.64 μg/L)were significantly higher than those in the S group, imparting intense fruity and fatty aromas to the wine [52,53]. These compounds belong to the category of medium and long chain fatty acid ethyl esters, which are mainly synthesized during the mid-to-late stages of alcoholic fermentation. Their formation is catalyzed by enzymes within yeast cells, among which alcohol acetyltransferase is the key enzyme responsible for catalyzing the esterification of fatty acids with ethanol [54]. SO_2_ may inhibit the activity of alcohol acetyltransferase, resulting in lower levels of fatty acid ethyl esters in the S group.

A total of seven types of acids were detected across both wine samples, with the TS100 group containing two more acid compounds than the S group. The total acid content was higher in the TS100 group (214.39 μg/L) than in the S group (149.7 μg/L). At low concentrations, appropriate levels of acidic compounds can enhance the aromatic profile of wine and help refine its overall bouquet, thereby adding complexity and depth to the wine body.

In summary, the addition of yeast polysaccharides alters the composition and concentration of aroma compounds in wine, resulting in prominent honey-like and fruity notes that distinguish it from wines traditionally treated with SO_2_. The overall aroma profile is richer, indicating that yeast polysaccharides are suitable for enhancing the aromatic quality of wine. While such modifications may offer a desirable sensory differentiation for consumers seeking novel wine styles, they also pose challenges for replicating traditional flavor expectations.

#### 3.6.2. Orthogonal Partial Least Squares Discriminant Analysis of Volatiles in Two Wines

To preliminarily explore whether differences existed in the aroma compounds between the two groups of wines, we performed PCA (Figure S1). The resulting biplot (PC1 = 89.4%, PC2 = 5.1%) revealed a clear separation along PC1. To further analyze the differences in volatile flavor compounds between the two wines, OPLS-DA was performed. As shown in Figure 5d, the S group is located on the negative side of the T [1] axis, while the TS100 group is positioned on the positive side, indicating a clear classification between the two wine groups. The VIP (Variable Importance in Projection) values quantify the contribution of each aroma compound to the sample differentiation. Compounds with VIP values greater than 1 were selected as key differential volatile components, as shown in Figure 5e, a total of 8 compounds with VIP > 1 were identified. Among them, Isopentanol, Phenethyl alcohol, and Phenethyl acetate had the highest VIP values, suggesting they are the most critical discriminating volatiles between the two wine samples.

#### 3.6.3. Wine Sensory Scoring

**Figure 6:**
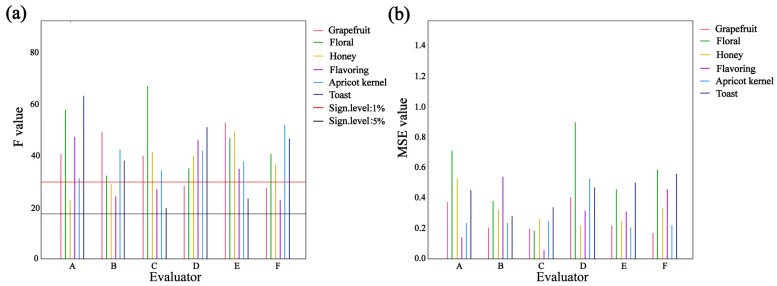
Tasting panel sensory ability attribute distinction and repetition ability. (**a**) The distinguishing ability of the evaluator; (**b**) The repeatability of the evaluator.

The Panel Check 1.4.2 software was used to evaluate the discrimination ability and repeatability of six panelists for the two wine samples. As shown in Figure 6, the F-values for all six descriptive terms evaluated by the panelists exceeded the 5% significance level, indicating effective differentiation. Evaluator A showed strong discrimination for the toasty aroma, while evaluator C demonstrated good discrimination for the floral aroma. Evaluator C also exhibited the highest overall repeatability, whereas evaluator D showed relatively lower repeatability for the floral descriptor, though the deviation did not exceed 0.020. Overall, the sensory panel displayed good consistency and was capable of providing reliable sensory evaluations of the wines.

**Figure 7:**
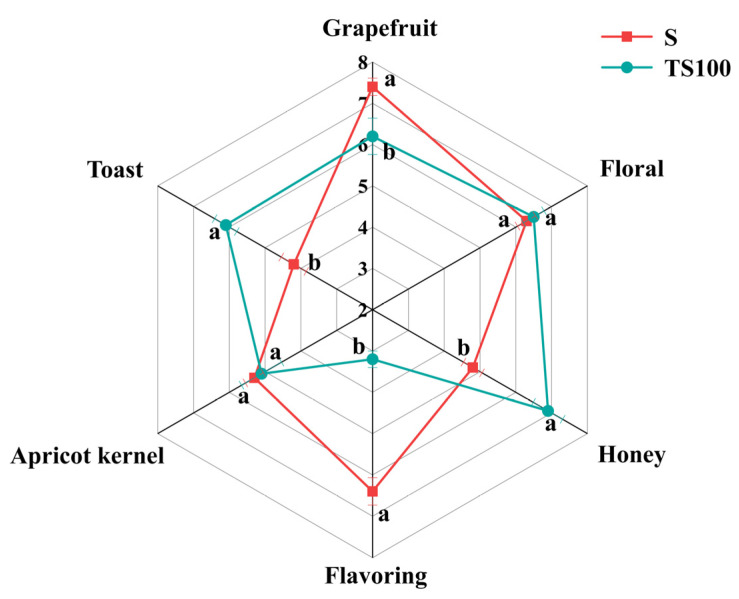
Aroma profiles of two groups of wines. Different superscript letters (a,b) for the same parameter denote significant differences (*p* < 0.05).

As shown in Figure 7, The six descriptors Grapefruit, Toast, Apricot kernel, Flavoring, Honey, and Floral were used for the sensory description of the wine [55,56,57,58]. The quantitative descriptive analysis (QDA) results indicate that yeast polysaccharides can influence the sensory characteristics of wine, enhancing honey and toasty aromas. Phenethyl alcohol brings out toast aromas [59]. In addition, phenolic volatiles such as furfural, 5-methylfurfural, and guaiacol are key contributors to toasty and baked aromas [60]. These compounds often exist in glycosidic bond forms, and mannoproteins can promote the hydrolysis of their precursors by glycosidases [36], thereby releasing more free, perceivable toasty aroma compounds. The increase in honey aroma may be attributed to the release of sweet-tasting amino acids such as proline and serine. In contrast, the spice aroma was significantly lower than that of the S group, likely because SO_2_ effectively protects key aroma compounds—such as thiols and some terpenes—that are highly sensitive to oxidation, a function that yeast polysaccharides cannot replicate. In summary, *Cabernet Sauvignon* wine treated with yeast polysaccharides exhibited richer toasty and honey aromas compared to conventionally SO_2_-treated wine. Its overall sensory profile was more harmonious, suggesting promising potential for the development of novel sulfur-free wines.

## 4. Conclusions

To address the potential health concerns associated with the use of SO_2_ in winemaking, this study explored the use of yeast polysaccharides as an alternative to SO_2_ and evaluated their effects on the basic physicochemical properties, antioxidant activity, flavor profile, and sensory quality of wines. The results demonstrated that the addition of YPs significantly improved the antioxidant capacity and phenolic retention of the wine. Wines treated with 100 mg/L YPs (TS100 group) exhibited the highest levels of polyphenols (1023.16 mg/L), total flavonoids (372.19 mg/L), and anthocyanins (340.79 mg/L), alongside enhanced free radical scavenging activity (DPPH: 86.25%, ABTS^+^: 82.23%), which were statistically comparable to those treated with SO_2_. In addition, the wine treated with YPs (TS100) exhibited a higher concentration of flavor compounds (10,458.99 μg/L) than the SO_2_-treated group (10,156.07 μg/L), showing elevated levels of ethyl esters such as ethyl caprylate and ethyl decanoate, which contributed to pronounced fruity and fatty aromas. Sensory analysis confirmed that YPs imparted favorable attributes including enhanced bread-like and honey notes, with a well-balanced flavor profile. Taken together, these findings indicate that yeast polysaccharides can effectively maintain wine quality, improve antioxidant properties, and provide distinct aromatic characteristics, thereby offering a promising natural alternative to sulfur dioxide in red wine production. However, the specific molecular mechanisms by which YPs exert their antioxidant effects remain unclear. Future research could further elucidate their interaction pathways with phenolic compounds, anthocyanins, and the mechanisms of free radical scavenging. Meanwhile, the broad-spectrum antimicrobial effects of YPs on the complex microbial community in wine have not yet been assessed, which is another potential direction for future work. These efforts would provide deeper theoretical evidence for the industrial application of yeast polysaccharides as an alternative to SO_2_ in winemaking.

## Figures and Tables

**Table 1:** Aroma descriptor references.

Serial Number	Aroma Descriptors	Reference Material
1	Grapefruit	*Le Nez du Vin~*Grapefruit
2	Floral	*Le Nez du Vin~*Linden flower
3	Honey	*Le Nez du Vin~*Honey
4	Flavoring	*Le Nez du Vin~*Cinnamon
5	Apricot kernel	*Le Nez du Vin~*Apricot kernel
6	Toast	*Le Nez du Vin~*Toast

**Table 2:** The physicochemical indexes of wines with different treatments.

Groups	Reducing Sugar (g/L)	Total Acid (g/L)	Soluble Solids (°Brix)	pH	Alcohol Content (% vol)
S	0.62 ± 0.08 ^a^	5.80 ± 0.15 ^b^	2.83 ± 0.11 ^de^	3.85 ± 0.08 ^a^	9.13 ± 0.21 ^a^
TS0	0.69 ± 0.03 ^ab^	6.04 ± 0.14 ^a^	3.24 ± 0.14 ^a^	3.87 ± 0.11 ^a^	8.22 ± 0.33 ^c^
TS50	0.64 ± 0.04 ^ab^	5.86 ± 0.07 ^b^	2.89 ± 0.09 ^cde^	3.89 ± 0.09 ^a^	8.87 ± 0.15 ^ab^
TS100	0.62 ± 0.03 ^ab^	5.77 ± 0.16 ^b^	2.77 ± 0.14 ^e^	3.86 ± 0.07 ^a^	9.14 ± 0.24 ^a^
TS150	0.63 ± 0.04 ^b^	5.73 ± 0.09 ^b^	3.02 ± 0.11 ^bcd^	3.84 ± 0.13 ^a^	8.61 ± 0.29 ^bc^
TS200	0.64 ± 0.02 ^ab^	5.84 ± 0.08 ^b^	3.14 ± 0.16 ^ab^	3.83 ± 0.06 ^a^	8.38 ± 0.11 ^c^
TS250	0.61 ± 0.02 ^ab^	5.76 ± 0.06 ^b^	3.09 ± 0.07 ^abc^	3.85 ± 0.09 ^a^	8.41 ± 0.17 ^c^

Different superscript letters (a–e) for the same parameter denote significant differences (*p* < 0.05).

## Data Availability

The original contributions presented in the study are included in the article/supplementary material, further inquiries can be directed to the corresponding author.

## Supplementary Materials

The following supporting information can be downloaded at: https://www.mdpi.com/article/10.3390/foods14183188/s1, Figure S1: Principal component analysis of volatile components in wines; Table S1: The content of volatile compounds (The concentration of the compounds reported in this table is relative to the response obtained for the internal standard).
