# Supplementary material for: Effect of Yeast Polysaccharides Replacing Sulfur Dioxide on Antioxidant Property and Quality Characteristics of Cabernet Sauvignon Wines
Source: Foods. 2025 Sep 12;14(18):3188. doi: 10.3390/foods14183188 (PMC12469342; doi:10.3390/foods14183188)
Supplement: Supplementary file 1 [file foods-14-03188-s001.zip › foods-3798920-supplementary.pdf]

Table S1. The content of volatile compounds. (The concentration of the compounds reported in this table is relative to the response obtained for the internal standard.)

Serial number	RI	Compounds	Concentration (μg/L)	
			S	TS100
1	1091	Isobutyl alcohol	252.43±3.23b	321.59±3.98a
2	1202	Isopentanol	5402.50±12.13a	4811.43±20.36b
3	1531	2,3-Butanediol	21.97±0.92a	20.61±0.79a
4	1719	3-Methylthiopropanol	13.98±0.84b	20.47±0.98a
5	1845	Phenethyl alcohol	2505.83±8.54b	2893.31±8.01a
6	1046	n-Propanol	32.90±1.14b	43.71±1.28a
7	1352	n-Hexyl alcohol	33.99±2.75a	35.47±1.34a
8	891	Ethyl acetate	252.43±2.78a	231.71±3.12b
9	1401	Ethyl octanoate	145.09±1.03b	297.63±1.64a
10	1583	Ethyl decanoate	355.92±1.86b	392.96±3.45a
11	1678	Ethyl trans-4-decenoate	-	222.64±5.9
12	1749	Phenethyl acetate	207.69±7.22b	338.08±9.62a
13	1107	Isoamyl acetate	195.64±2.19a	178.34±3.11b
14	1772	Ethyl laurate	63.91±3.3b	91.32±1.94a
15	2242	Ethyl hexadecanoate	138.89±2.24a	90.21±1.76b
16	1218	Ethyl hexanoate	128.22±0.96b	134.12±2.12a
17	1646	3-Methylbutyl octanoate	39.24±0.68a	26.34±1.12b
18	1970	3-Methylbutyl decanoate	-	45.35±1.66
19	1801	Phenethyl butyrate	-	12.37±0.54
20	2173	2-Phenylethyl octanoate	-	4.12±0.18
21	1720	Ethyl phenylacetate	7.99±0.20a	5.89±0.98b
22	2243	Ethyl palmitate	51.92±0.73a	20.61±1.06b
23	3247	Ethyl oleate	11.98±1.15a	8.25±0.79b
24	2385	Ethyl linoleate	13.98±0.83a	12.37±0.77a
25	1955	Acetic acid	43.86±1.02b	53.6±0.86a
26	1579	2-Methylhexanoic acid	31.95±2.81b	41.23±1.93a
27	1493	Hexanoic acid	17.97±1.06b	24.74±3.30a
28	2063	Octanoic acid	39.94±0.69b	61.84±2.51a
29	2170	Nonanoic acid	-	4.12±1.25
30	2269	Decanoic acid	15.98±0.32b	24.74±2.77a
31	1980	9-Decenoic acid	-	4.12±0.36
32	2192	2,4-Di-tert-butylphenol	129.80±5.8	-

Different superscript letters (a-b) for the same parameter denote significant differences ($p < 0.05$).

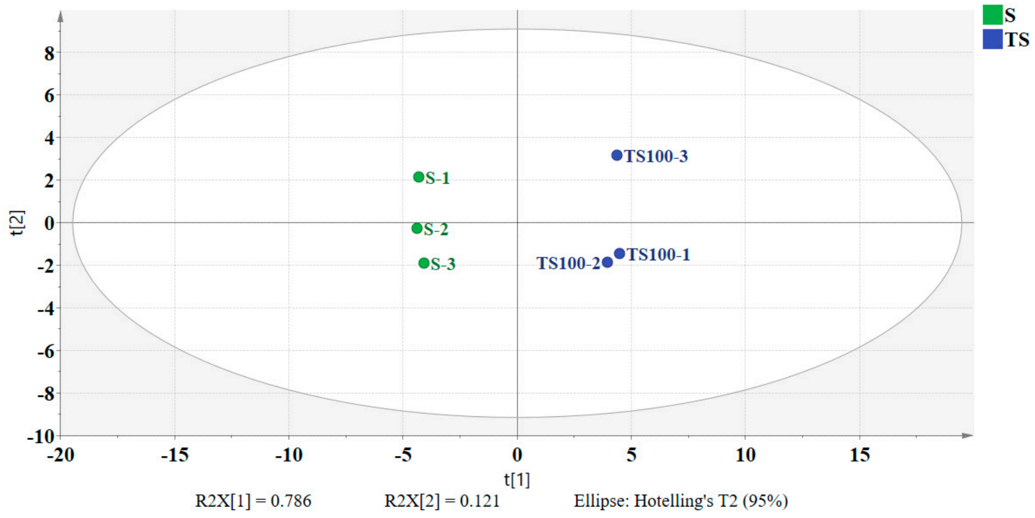

Figure S1. Principal component analysis of volatile components in wines.
